# Quantitative analysis of contrast enhanced spectral mammography grey value for early prediction of pathological response of breast cancer to neoadjuvant chemotherapy

**DOI:** 10.1038/s41598-021-85353-9

**Published:** 2021-03-15

**Authors:** Dong Xing, Ning Mao, Jianjun Dong, Heng Ma, Qianqian Chen, Yongbin Lv

**Affiliations:** 1 Department of Radiology, Yantai Yuhuangding Hospital, Affiliated Hospital of Qingdao University, No. 20 Yuhuangding East Road, Yantai, 264000 Shandong People’s Republic of China; 2 GE Healthcare, Institute of Precision Medicine, No. 1 Huatuo Road, Shanghai, 201203 People’s Republic of China

**Keywords:** Radiography, Cancer imaging, Breast cancer

## Abstract

A quantitative analysis of contrast-enhanced spectral mammography (CESM) enhancement was conducted for the early prediction of the pathological response after neoadjuvant chemotherapy (NAC). Retrospective analysis of the data of 111 patients was conducted, and all of them underwent NAC in our hospital and surgical resection after the end of all cycles from January 2018 to May 2019. They were divided into pathological complete response (PCR) and non-PCR groups. We determined whether a statistical difference in the percentage of CESM grey value reduction (ΔCGV) was present in the PCR and non-PCR groups and whether a statistical difference was observed in the diagnostic efficiency of craniocaudal (CC) and mediolateral oblique (MLO) view subtraction images. Independent sample t-test was used to compare different groups, the receiver operating characteristic (ROC) curve was used to compare the diagnostic efficacy of CC and MLO for pathological response after NAC, and the Delong test was used to compare the area under the ROC curve (AUC). Statistical significance was considered at P < 0.05. A statistical difference was observed in the ΔCGV in the PCR and non-PCR groups. No statistical difference was observed in the AUCs of CC and MLO view subtraction images. The ΔCGV can be used as a quantitative index to predict PCR early, and no statistical difference was observed in the diagnostic efficacy of CC and MLO view subtraction images.

## Introduction

Neoadjuvant chemotherapy (NAC) has become a standard treatment for patients with locally advanced breast cancer and is increasingly being used in treating patients with surgically treatable breast cancer^1,2^. NAC can reduce the size of primary tumors, thereby increasing the possibility of breast-conserving surgery^3^. Breast cancer’s pathological response to NAC is associated with the patient’s prognosis^4^, and pathological complete response (PCR) can significantly improve a patient’s disease-free and overall survival rates^5,6^. However, the pathological complete response rate is low (10%–31%), with the total response rate ranging from 69% to100%^7^. Primary tumors with poor treatment effectiveness can be found through the early assessment of breast cancer’s response to NAC, which is conducive to changing the treatment plan in time and arranging the time for operation reasonably, so as to avoid the toxic side effects of chemotherapy and reduce treatment costs.

Imaging technology is an important method to evaluate breast cancer’s response to NAC. At present, mammography, ultrasound, and MRI are used to evaluate pathological responses after NAC, with MRI being the most accurate^8^. However, MRI has a long examination time, produces loud noise, and is expensive. Thus, the use of MRI has certain restrictions. Contrast-enhanced spectral mammography (CESM) is a new imaging technique that uses contrast agents to conduct examinations on the basis of digital mammography, with high- and low-energy exposures after intravenous contrast agents, which are processed to obtain low-energy and subtraction images. The diagnostic efficacy of CESM low-energy images is equivalent to that of digital mammography^9^. The subtraction images display areas of contrast enhancement reflecting the vascularity of breast lesions. The diagnostic accuracy of CESM is comparable to breast MRI^10,11^, and its specificity is even better than that of MRI, especially in patients with contraindications to MRI examinations (e.g., patients with pacemaker implantation and claustrophobia).

Malignant breast tumors are accompanied by neovascularization in the tumor during growth. The growth, infiltration, and distant metastasis of malignant breast tumors are dependent on these new blood vessels ^12^. Effective chemotherapy can reduce tumor neovascularization and microvascular permeability to achieve therapeutic effects^13^. This phenomenon is shown in CESM subtraction images as a reduction in lesion enhancement^14^. The grey value can quantify the degree of enhancement of CESM^15^. Thus, the present study aimed to explore the effects of the CESM grey value (CGV) reduction percentage in the early prediction of NAC pathological responses.

## Materials and methods

This was a retrospective study approved by the ethics committee of Yantai Yuhuangding Hospital (IRB approval number: 2019391), and informed consent was not required. The study followed the principles of the Declaration of Helsinki with voluntary participation. The data were analyzed and handled in an anonymous format. We adhered to relevant guidelines and regulations in all experiments.

### Patients

This study included breast cancer patients who underwent NAC between January 2018 and May 2019 in our hospital. The information of patients was obtained from electronic medical records. The inclusion criteria included the following: (1) before NAC, patients were confirmed to have invasive breast cancer by core needle biopsy pathology; (2) each patient received CESM two times: the first time was before NAC, and the second time was two weeks after the second cycle of NAC and before the start of the third cycle; (3) surgical resection was performed after the end of NAC; and (4) postoperative pathological sections were satisfactory, and accurate histopathological evaluation was obtained. The exclusion criteria are as follows: (1) patients who have previously received hormone, chemotherapy, and radiotherapy before NAC; (2) patients who have changed treatment options during NAC; (3) patients with known or suspected allergy to iodine contrast agents or other contrast agents; (4) patients with known or suspected renal insufficiency; and (5) women who were pregnant, preparing for pregnancy or breastfeeding.

### Treatment scheme

All patients received eight cycles of chemotherapy. NAC consists of two plans. All patients with human epidermal growth factor receptor type 2 (HER2) negative tumors were treated with four cycles of epirubicin and cyclophosphamide, followed by four cycles of docetaxel. Patients with HER2-positive tumors were treated with four cycles of epirubicin and cyclophosphamide, followed by four cycles of docetaxel and trastuzumab. Surgical excision was performed after the end of the full cycle of NAC.

### Imaging technology

CESM was performed using the Senographe Essential all-digital mammography system (GE Healthcare, Inc, Princeton, USA). Iohexol (containing 350 mg/ml of iodine, Beilu Pharmaceutical Co, Ltd. Beijing, China) was used as the contrast agent at a dose of 1.5 ml/kg and injected into the upper arm vein through a high-pressure syringe at a flow rate of 3 ml/s. After injection for approximately 2 min, the imaging was projected in the following order: the craniocaudal (CC) view of the breast on the affected side, the CC view of the breast on the unaffected side, the mediolateral oblique (MLO) view of the breast on the unaffected side, and finally the MLO view of the breast on the affected side. The radiographic process of each patient was completed in 7 min. In radiography, a low-energy and a high-energy exposure was obtained continuously within 1.5 s of a compression. Two images, namely a low-energy image and a subtraction image, were obtained for each position on the workstation.

### Analysis of images

**Figure 1:**
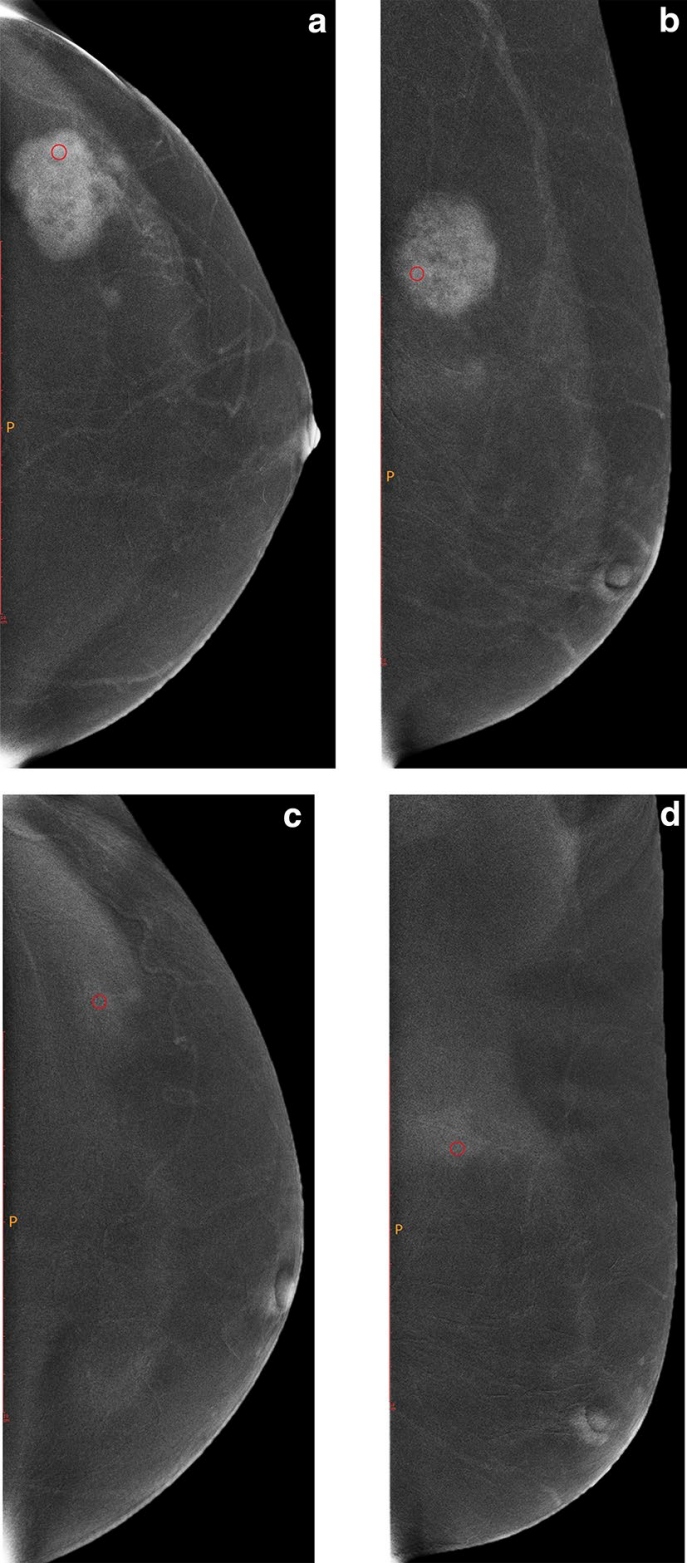
Female, 51 years old, with lesion located in the outer upper quadrant of the left breast. **(a,b)** are the subtraction images of CC and MLO views of CESM before NAC, and **(c,d)** are the subtraction images of CC and MLO views of CESM after two cycles of NAC, respectively. The CC and MLO view grey value reduction percentages were 40.27% and 16.13%, respectively. Before surgery, core needle biopsy confirmed that the patient had invasive breast cancer. After surgical resection, the pathological results showed no invasive cancer components with MP Grade 5.

**Figure 2:**
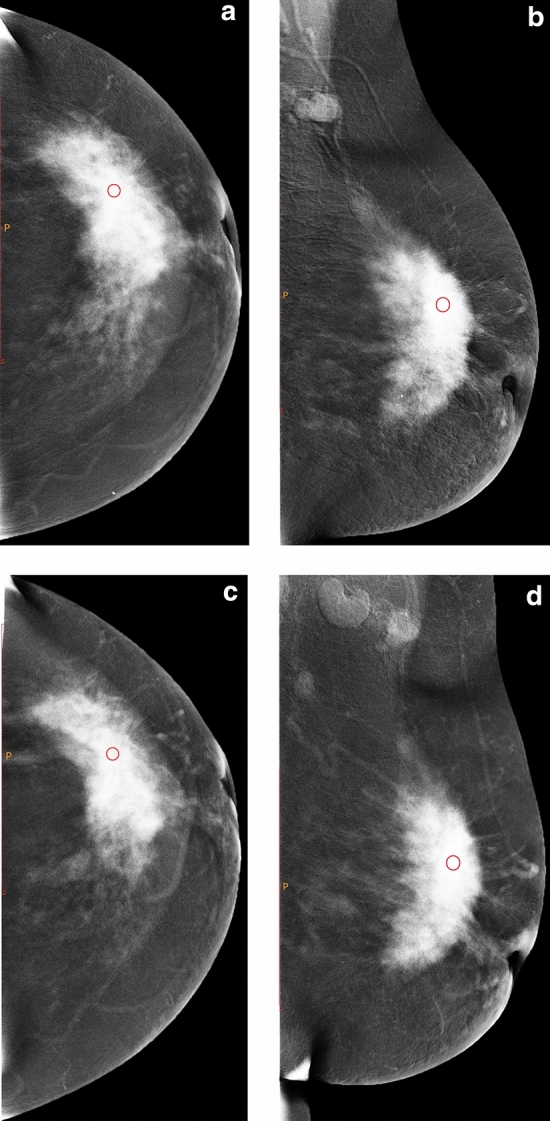
Female, 49 years old, with lesion located in the left outer quadrant. a and b are the subtraction images of CC and MLO views of CESM before NAC, and c and d are the subtraction images of CC and MLO views of CESM after two cycles of NAC, respectively. The CC and MLO view grey value reduction percentages were 4.02% and 4.95%, respectively. Before surgery, core needle biopsy confirmed that the patient had invasive breast cancer. After surgical resection, the pathological results showed invasive ductal carcinoma Grade II with MP Grade 3.

A radiologist with 10 years of experience manually outlined the region of interest (ROI) on the CESM subtraction image (including the CC and MLO views). The ROI was placed in the area with the most evident lesion enhancement (highest grey value). The area of liquefaction necrosis should be avoided. The ROI was 0.3–0.5 cm^2^ in size. Each patient underwent CESM twice (the first time was before NAC, and the second time was two weeks after the second cycle of NAC and before the start of the third cycle). For the subtraction images of the same patient on the same projection position, the size of the ROI that was outlined twice should be the same or close (Figs. 1 and 2). The grey value in the ROI was calculated and exported through ImageJ 1.52p software (https://imagej.en.softonic.com/). The ROI was placed three times in the most evident area of the enhancement, thereby taking the average of the three results as the grey value of the ROI. For patients with multifocal disease, grey values were only measured in areas with the highest degree of enhancement. All patients had unilateral lesions.

The radiologists were unaware of the pathological results.

### Calculation of grey value reduction percentages (ΔCGV)

The ΔCGV can be calculated as follows: ΔCGV = (grey value before NAC − grey value after two cycles of NAC)/grey value before NAC × 100%.

### Histopathological assessment

The pathological response of NAC on surgical specimens was evaluated by an experienced breast pathologist on the basis of the Miller-Payne (MP) grading system. The histopathological slices after surgery and puncture histological slices before chemotherapy were compared, and the pathological response was divided into five grades according to the regression of tumor cells under the microscope on the basis of the MP grading system. For Grade 1, no change was observed in infiltrating cancer cells, or several changes were only observed in individual cancer cells; the overall number of cancer cells did not decrease. For Grade 2, the number of infiltrating cancer cells decreased slightly, but the total number was still high, and the number of cancer cells decreased by no more than 30%. For Grade 3, the infiltrating cancer cells decreased by 30–90%. For Grade 4, the infiltrating cancer cells were significantly reduced by more than 90%, with only small clusters of cancer cells or individual cancer cells remaining. For Grade 5, no infiltrating cancer cells were observed in the original tumor bed, but ductal carcinoma in situ may exist. PCR was defined as Grade 5 and Grades 1–4 were defined as non-PCR.

The expression of estrogen receptors (ER), progesterone receptors (PR), and HER2 was evaluated. HER2 expression was initially rated as 0, 1 + , 2 + , or 3 + through immunohistochemical staining. Tumors rated 3 + were classified as HER2 positive, and those rated 0 or 1 + were classified as HER2 negative. In tumors with a score of 2 + , the HER2 gene status of the tumor tissue was detected using fluorescence in situ hybridization technology, and HER2 was considered positive if the result was HER2 gene amplification. Hormone receptor (HR) positive indicated ER and/or PR positive (≥ 10% nuclear staining). The tumor subtypes are classified as follows: Luminal A (HR + /HER2−, Ki67 low), Luminal B HER2 negative or HER2 positive (HR + /HER2−, Ki67 high or HR + /HER2 +), Triple negative (HR−/HER2−), and HER2 enriched (HR−/HER2 +).

### Statistical analysis

The data were statistically described and analyzed using SPSS 22.0 software. The measurement data were described by mean ± standard deviation ($\stackrel{-}{x}$±s), and the intergroup comparison was tested using independent sample t-test. The diagnostic value of NAC response was evaluated using the receiver operating characteristic (ROC) curve. Statistical significance was considered at P < 0.05.

### Ethical statement

All authors confirm that all methods are in accordance with the relevant guidelines and regulations in the manuscript documentation.

## Results

### Patient characteristics and response to NAC

A total of 111 patients were included in the study (all women, age range of 26–67 years, mean age of 51 ± 9.1 years). Out of the 111 patients, 32 were PCR, and 79 were non-PCR. The MP grading results for surgical specimens are as follows: 5 cases of Grade 1, 20 cases of Grade 2, 33 cases of Grade 3, 21 cases of Grade 4, and 32 cases of Grade 5. All patients’ pathological types and tumor subtypes are listed in Table 1.

**Table 1:** Patients demographic characteristics.

No. of women	111
**Age (year)**	
> 40 ≤ 40	93 18
**Breast density**	
BI-RADS A BI-RADS B BI-RADS C BI-RADS D	11 25 56 19
**Menopausal status**	
Premenopausal Postmenopausal	62 49
**Pathological type**	Number of cases
Infiltrating carcinoma	50
Infiltrating ductal carcinoma	56
Infiltrating papillary carcinoma	1
Infiltrating lobular carcinoma	2
Infiltrating lobular carcinoma with infiltrating ductal carcinoma	1
Mucinous carcinoma	1
**ER status**	
Negative	29
Positive	82
**PR status**	
Negative	42
Positive	69
**HER2 status**	
Negative	70
Positive	41
**Ki67**	
≥ 20% < 20%	64 47
**Tumor subtypes**	
Luminal A	51
Luminal B	33
HER2-enriched	15
Triple negative	12

### Diagnostic performances in determining PCR

Before NAC, the differences were not statistically significant between the CC and MLO view grey values of the PCR and non-PCR groups (P > 0.05). After two cycles of chemotherapy, the grey values of the CC and MLO views of the PCR group were lower than those of the non-PCR group, and the ΔCGV of the CC and MLO views was higher than that of the non-PCR group. The difference was statistically significant (P < 0.05, Table 2).

**Table 2:** Comparison of the percentage of grey value reduction between PCR and non-PCR groups.

	PCR (*n* = 32)	non-PCR (*n* = 79)	*t*	*P*
CC view grey value before chemotherapy	123.97 ± 38.81	132.40 ± 37.48	− 1.063	0.290
After two cycles of chemotherapy	73.48 ± 9.72	105.43 ± 34.01	− 5.216	< 0.001
Reduction percentage (%)	36.53 ± 17.54	19.63 ± 13.64	5.430	< 0.001
MLO view grey value before chemotherapy	116.71 ± 27.98	129.87 ± 34.40	− 1.920	0.057
After two cycles of chemotherapy	80.70 ± 17.25	106.59 ± 32.33	− 4.282	< 0.001
Reduction percentage (%)	28.90 ± 14.23	17.01 ± 14.46	3.942	< 0.001

The test variables were the CC and MLO view ΔCGV, and the ROC curve was fitted with pathological response as the state variable. The results showed that the ΔCGV of the CC view (AUC = 0.776) and MLO view (AUC = 0.733) had significant diagnostic value (P < 0.001). When the CC view ΔCGV had a cut-off value of > 26.41, it had the best diagnostic effect on pathological response, with a sensitivity and specificity of 75.00% and 72.15%, respectively. When the MLO view ΔCGV had a cut-off value of > 13.59, it had the best diagnostic effect on pathological response, with a sensitivity and specificity of 81.25% and 51.90%, respectively (Table 3).

**Table 3:** Analysis of the diagnostic value of CC and MLO views grey value reduction percentages.

Photography position	AUC	95% CI	P	Cut-off	Sensitivity	Specificity
CC view	0.776	0.679–0.855	0.001	> 26.41	75.00	72.15
MLO view	0.733	0.632–0.819	< 0.001	> 13.59	81.25	51.90

### Comparison of the diagnostic efficacy of different photography positions

**Figure 3:**
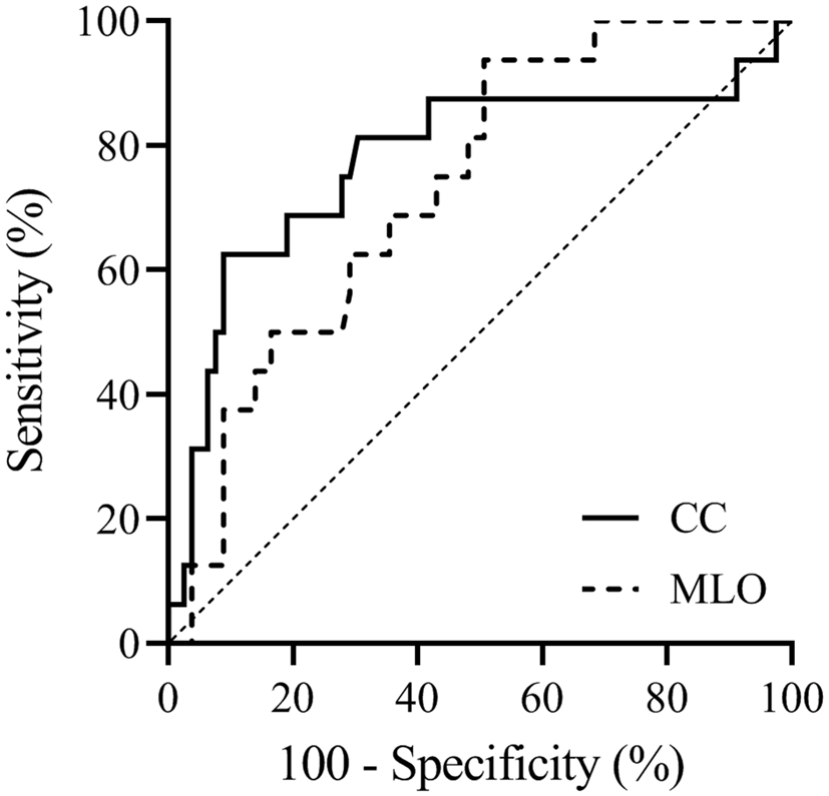
Diagnostic efficiency ROC curve of CC and MLO view grey value reduction percentages.

The comparison of the AUCs of the CC and MLO view ΔCGV showed that the difference was not statistically significant (Delong test, P = 0.460) (Fig. 3).

## Discussion

In this study, the value of quantitative analysis of the CGV in the early prediction of NAC pathological responses was explored. The results of this study showed that there was a significant difference in the ΔCGV in the PCR and non-PCR groups after the second cycle of NAC, indicating that the ΔCGV can be used as a quantitative index to predict PCR early. The ΔCGV of CC and MLO view subtraction images had significant diagnostic value. No statistical difference was observed in the AUC of the two views, thereby indicating that the two predicted PCR had the same diagnostic efficiency.

Various studies have compared the roles of CESM and MRI in evaluating NAC responses. Patel et al.^8^ compared the size of residual breast cancer on post-NAC CESM and MRI with surgical specimens and showed that CESM and MRI are comparable in assessing residual breast cancer after NAC. Iotti et al. ^16^ asked patients to undergo CESM and breast MRI before NAC, during NAC, and after the end of NAC. Then, after the end of NAC, they compared the measurement results of CESM and MRI with the surgical specimens according to the RECIST 1.1 standard. The result showed that CESM is highly correlated with the size of the lesion measured by MRI, which leads to the following conclusion. CESM is at least as reliable as MRI when evaluating responses to NAC and can be used as an alternative if a contraindication in performing MRI exists or its availability is limited. However, previous studies only assessed the size of the tumor, and had several limitations. First, the enhancement mode of breast cancer on the CESM subtraction image was divided into mass and non-mass enhancement, and the size of the lesions for non-mass enhanced lesions was difficult to measure accurately. Second, the two modes of tumor cell regression were observed after NAC. These modes include concentric shrinkage, wherein the tumor shrinks concentrically, and non-concentric shrinkage, wherein the tumor shrinks into multiple lesions^17^, and the lesions split into scattered tumor islands or fragments, thereby increasing the difficulty of accurately measuring the size of the lesions^18^. Studies conducted by Patel et al.^8^ and Iotti et al.^16^ also suggested that CESM and MRI overestimate or underestimate the size of lesions, respectively. Third, the overall reduction of tumor cells after chemotherapy is not always reflected by a decrease in tumor size because even if tumor cells are destroyed, fibrous stroma still exists^19^. These limitations make the prediction of post-NAC pathological responses by tumor size, especially early prediction, difficult.

Some studies have shown that the changes in angiogenesis during NAC treatment predate the variations in tumor size^20^. To avoid the limitations caused by tumor size as the evaluation criterion, this study adopted an approach to quantify the degree of CESM enhancement to predict NAC response early. At present, studies on the quantitative analysis of the degree of CESM enhancement to predict NAC response are extremely limited. Moustafa et al.^14^ reported that the quantitative analysis of the enhancement of CESM can objectively and accurately evaluate the response of breast cancer to chemotherapy and can be used as an alternative to subjective techniques for preoperative examination. Several differences were observed in the present study. First, Moustafa et al. divided the subjects into responders (MP grades III, IV, and V) and non-responders (MP grades I and II), which is different from the results of our study. In the present study, the subjects were divided into the PCR and non-PCR groups because the main purpose of NAC is to reduce the size of lesions and achieve PCR. Breast cancer patients who received PCR had a significantly lower risk of recurrence or death than those with residual disease, regardless of neoadjuvant therapy^21^. If patients can receive PCR before surgery, they may not need surgery after NAC in the future^22^. Thus, PCR should be predicted using imaging methods. Second, Moustafa et al. chose to perform CESM before and after NAC, a method that, while evaluating breast cancer’s response to NAC after the end of NAC, also missed the opportunity to change treatment options. A complete NAC course takes several months, and the early evaluation of NAC response can timely identify tumors that are ineffective or even insensitive to chemotherapy, thereby avoiding unnecessary chemotherapy toxicity, rationally scheduling surgery, and saving medical costs. In this study, the chemotherapy response after the second cycle of NAC was selected for an early assessment, and our results should be more clinically instructive.

No unified standard on which cycle of NAC can be predicted by imaging examinations is currently in place. Padhani et al.^23^ reported that the range of volume transfer constant (Ktrans) can predict pathological responses after two cycles of NAC. Ah-See et al.^24^ indicated that Ktrans and exchange rate constant (Kep) are closely related to pathological responses after two cycles of NAC. Whether pharmacokinetic parameters for dynamic contrast-enhanced MRI (DCE-MRI) can predict pathological responses after one cycle of NAC is currently debatable. Li et al.^25^ reported that Kep can predict therapeutic responses after the first cycle of NAC, while Cho et al.^26^ believed that the parameters of DCE-MRI (i.e., Ktrans, Kep, and extravascular extracellular space volume) cannot recognize pathological responses early after the first cycle of NAC. DCE-MRI parameters reflect blood flow, vascular density, and vascular permeability^27^. Thus, the changes in pharmacokinetic parameters after NAC reflect the response of tumor blood vessels to therapeutic effects^28^. The time after the second cycle of NAC was regarded as a point in time to evaluate the pathological response. The results of the present study showed that quantitative analysis of the CGV after the second cycle of NAC has certain value for the early prediction of the pathological response.

Although MRI is currently the best method^8^ to evaluate the pathological response of NAC, it is limited in use, and also expensive. For the diagnosis of the disease in the breast, the accuracy of CESM is comparable to that of breast MRI, with the former having a better specificity than the latter ^10^ ^11^. CESM is also cheaper, more accessible, and more tolerable than breast MRI, thereby making it a suitable alternative for patients. The results of this study further showed that CESM has certain value for the early prediction of NAC pathological response, especially when the application of breast MRI is limited, and it can be used as an alternative to MRI.

Despite the findings, this study has limitations. First, due to the small sample size, each tumor subtype cannot be evaluated separately when quantitatively analyzing the CGV. Studies have reported that different tumor subtypes have varying responses to NAC treatment^29^.Thus, this research method may have different results for different tumor subtypes, which needs to be further studied by expanding the sample size in the future. Second, although our research results show that the diagnostic efficacy of CC and MLO view is not statistically different, this is not enough to conclude that both positions have the same diagnostic efficacy. It may be due to the small sample size. This result also requires further study by expanding the sample size.Third, The results of this study show that the specificity of MLO view is low. We believe that expanding the sample size to study different tumor subtypes separately may improve specificity. Fourth, a few breast lesions lack CESM enhancement, including invasive carcinoma and ductal carcinoma in situ^30^. This conclusion limits the application of the results of this study. Fifth, this study only distinguishes PCR from non-PCR by the single factor of grey value, which inevitably has limitations. Radiomics is a multi- features analysis related to clinical results, and tremendous progress has been made in recent years. Studies have pointed out that the use of computer-aided diagnosis systems can distinguish benign and malignant microcalcification clusters of the breast^31^. Research on CESM radiomics pointed out that radiomic analysis of tumor features extracted from CESM images can distinguish benign and malignant breast lesions^32^ and further predict histological outcomes and particular subtypes of tumors^33^. The use of radiomic analysis in this study should be of great significance.

## Conclusion

The results of this study showed that the quantitative analysis of the CGV after the second cycle of NAC has certain value for the early prediction of pathological response.

## Data Availability

The data generated in the current study are available from the corresponding author on reasonable request.
